# Supplementary material for: Gene regulatory network inference using mixed-norms regularized multivariate model with covariance selection
Source: PLoS Comput Biol. 2023 Jul 31;19(7):e1010832. doi: 10.1371/journal.pcbi.1010832 (PMC10414675; doi:10.1371/journal.pcbi.1010832)
Supplement: S1 Table — The reported results are from the DREAM5 challenge and correspond to the best (i.e. overall score) inference methods that participated in the challenge. Since results obtained using the R package “precrec” were slightly different from those of the challenge (cf. Table 1), we sought to include the latter here to have a comprehensive assessment and to avoid misinterpretation of the current results. (PDF) [file pcbi.1010832.s002.pdf]

S1 Table. Comparison of model performance using area under the ROC curve (AUROC) and area under the precision-recall curve (AUPR) on DREAM5 data sets.

**Winner of the challenge**									
**Methods**	***In silico*** **(Network 1)**		***E. coli*** **(Network 3)**		***S. cerevisiae*** **(Network 4)**		**Score**		
	**AUROC**	**AUPR**	**AUROC**	**AUPR**	**AUROC**	**AUPR**	**AUROC**	**AUPR**	**Overall**
GENIE3	**0.815**	0.291	0.617	0.093	0.518	0.021	0.638	0.082	0.360
ANOVerence	0.780	0.245	**0.671**	**0.119**	**0.519**	**0.022**	**0.647**	**0.086**	**0.366**
TIGRESS	0.782	**0.301**	0.595	0.069	0.517	0.020	0.621	0.074	0.348

The reported results are from the DREAM5 challenge and correspond to the best (i.e. overall score) inference methods that participated in the challenge. Since results obtained using the R package "precrec" were slightly different from those of the challenge (cf. Table 1 **??**), we sought to include the latter here to have a comprehensive assessment and to avoid misinterpretation of the current results.
