# Supplementary material for: Gene regulatory network inference using mixed-norms regularized multivariate model with covariance selection
Source: PLoS Comput Biol. 2023 Jul 31;19(7):e1010832. doi: 10.1371/journal.pcbi.1010832 (PMC10414675; doi:10.1371/journal.pcbi.1010832)
Supplement: S1 Text — The Text includes detailed explanations on how to derive: (1) The matrix of regression coefficients B, as the solution to a special case of Sylvester equation, (2) The special cases of the L1L2,1 and L2L2,1 solutions as well as the precision matrix Ω as the solution to a special form of algebraic Riccati equation. (PDF) [file pcbi.1010832.s004.pdf]

S1 Text
Supplementary methods for: Gene regulatory network inference using mixed-norms regularized multivariate model with covariance selection

Alain J. Mbebi[1,2], Zoran Nikoloski[1,2*]

1 Bioinformatics Department, Institute of Biochemistry and Biology, University of Potsdam, Karl-Liebknecht-Str. 24-25, 14476 Potsdam-Golm, Germany
2 Systems Biology and Mathematical Modeling Group, Max Planck Institute of Molecular Plant Physiology, Am Mühlenberg 1, 14476 Potsdam-Golm, Germany

*nikoloski@mpimp-golm.mpg.de

Method 1: B as the solution to a special case of Sylvester equation

Considering the following inohomogeneous Sylvester equation [1] in term of $\mathbf{B}$:

$$\mathbf{X}^T\mathbf{X}\mathbf{B} + n\lambda_2\mathbf{B}\mathbf{C}\boldsymbol{\Omega}_0^{-1} = \mathbf{X}^T\mathbf{Y} \tag{S1}$$

and defining $\mathbf{X} = \mathbf{U}_1\boldsymbol{\Gamma}_1\mathbf{V}_1^T$ as the singular value decomposition (SVD) of $\mathbf{X}$ then,

$$\begin{aligned}\text{Eq }(S1) \Rightarrow & \mathbf{V}_1\boldsymbol{\Gamma}_1^T\mathbf{U}_1^T\mathbf{U}_1\boldsymbol{\Gamma}_1\mathbf{V}_1^T\mathbf{B} + n\lambda_2\mathbf{B}\mathbf{C}\boldsymbol{\Omega}_0^{-1} = \mathbf{X}^T\mathbf{Y} \\ \Rightarrow & \boldsymbol{\Gamma}_1^T\boldsymbol{\Gamma}_1(\mathbf{V}_1^T\mathbf{B}) + n\lambda_2(\mathbf{V}_1^T\mathbf{B})\mathbf{C}\boldsymbol{\Omega}_0^{-1} = \mathbf{V}_1^T\mathbf{X}^T\mathbf{Y}.\end{aligned} \tag{S2}$$

By further making the following change of variables:

$$\begin{cases}\tilde{\mathbf{B}} & = \mathbf{V}_1^T\mathbf{B} \in \mathbb{R}^{n\times s} \\ \mathbf{S} & = \mathbf{V}_1^T\mathbf{X}^T\mathbf{Y} \in \mathbb{R}^{n\times s} \\ \mathbf{K} & = \mathbf{C}\boldsymbol{\Omega}_0^{-1} \in \mathbb{R}^{s\times s} \\ \boldsymbol{\Gamma}_1^T\boldsymbol{\Gamma}_1 & = \text{diag}(\gamma_1, \gamma_2, \cdots, \gamma_n) \in \mathbb{R}^{n\times n}\end{cases} \tag{S3}$$

Eq (S2) becomes $\boldsymbol{\Gamma}_1^T\boldsymbol{\Gamma}_1\tilde{\mathbf{B}} + n\lambda_2\tilde{\mathbf{B}}\mathbf{K} = \mathbf{S}$, or equivalently

$$\begin{bmatrix}\gamma_1\tilde{b}_{11} & \cdots & \gamma_1\tilde{b}_{1s} \\ \vdots & \vdots & \vdots \\ \gamma_n\tilde{b}_{n1} & \cdots & \gamma_n\tilde{b}_{ns}\end{bmatrix} + n\lambda_2\begin{bmatrix}\tilde{b}_{11} & \cdots & \tilde{b}_{1s} \\ \vdots & \vdots & \vdots \\ \tilde{b}_{n1} & \cdots & \tilde{b}_{ns}\end{bmatrix}\mathbf{K} = \begin{bmatrix}s_{11} & \cdots & s_{1s} \\ \vdots & \ddots & \vdots \\ s_{n1} & \cdots & s_{ns}\end{bmatrix} \tag{S4}$$

If the problem in Eq (S4) above admits a solution, then it must hold that for every row $1 \le i \le n$,

$$\tilde{\mathbf{b}}^i\gamma_i\mathbf{I}_s + n\lambda_2\tilde{\mathbf{b}}^i\mathbf{K} = \mathbf{s}^i \Rightarrow \tilde{\mathbf{b}}^i = \mathbf{s}^i\left[\gamma_i\mathbf{I}_s + n\lambda_2\mathbf{K}\right]^{-1}, \tag{S5}$$

where $\tilde{\mathbf{b}}^i = \begin{bmatrix}\tilde{b}_{i1}, & \tilde{b}_{i2}, & \cdots, & \tilde{b}_{is}\end{bmatrix}$ and $\mathbf{s}^i = \begin{bmatrix}s_{i1}, & s_{i2}, & \cdots, & s_{is}\end{bmatrix}$ are the ith row of $\tilde{\mathbf{B}}$ and $\mathbf{S}$ respectively.

It only remains to show that every $\tilde{\mathbf{b}}^i$ exists and is unique, or equivalently, show the existence of $\mathbf{Z}_i^{-1} = [\gamma_i \mathbf{I}_s + n\lambda_2 \mathbf{K}]^{-1}$. From the matrix inversion lemma [2] and recalling that $\mathbf{K} = \mathbf{C}\boldsymbol{\Omega}_0^{-1}$, we know that, $\mathbf{Z}_i^{-1}$ exists if $\gamma_i \mathbf{I}_s$ and $\mathbf{C} + n\lambda_2 \mathbf{C}\boldsymbol{\Omega}_\mathbf{0}\mathbf{C}$ are non singular. As a diagonal matrix with strictly positive entries, $\mathbf{C}$ is symmetric and positive definite (PD). Columns of $\mathbf{C}$ being linearly independent and $\boldsymbol{\Omega}_\mathbf{0}$ symmetric PD imply that $n\lambda_2 \mathbf{C}\boldsymbol{\Omega}_\mathbf{0}\mathbf{C}$ is also symmetric and PD. This is sufficient to say that their sum is PD and therefore, non singular. From the definition of the SVD, we know that $\gamma_i \geq 0$. This means that, if $\gamma_i = 0$, the determinant of $\mathbf{K} = \mathbf{C}\boldsymbol{\Omega}_0^{-1} > 0$, otherwise, the non-singularity of the diagonal matrix $\gamma_i \mathbf{I}_s$ is guarantied when $\gamma_i > 0$ and this ensures the existence of $\mathbf{Z}_i^{-1}$. Recalling the change of variable $\tilde{\mathbf{B}} = \mathbf{V}_1^T \mathbf{B}$, we finally obtain $\mathbf{B} = \mathbf{V}_1 \tilde{\mathbf{B}} \in \mathbb{R}^{p \times s}$ and subsequently refer to this as $\mathrm{L}_1\mathrm{L}_{2,1}$- solution.

Method 2: Special cases of the $\mathbf{L}_1\mathbf{L}_{2,1}$ and $\mathbf{L}_2\mathbf{L}_{2,1}$ solutions

In this section, we show that under certain conditions, the proposed mixed $\mathrm{L}_1\mathrm{L}_{2,1}$-norm and $\mathrm{L}_2\mathrm{L}_{2,1}$-norm regularized multivariate regression and covariance selection models can be viewed as a generalization of the following:

i. When $\mathbf{C} = \mathbf{I}_s$, the $\mathrm{L}_{2,1}$-norm regularization on the regression coefficient matrix becomes $\mathrm{Tr}(\mathbf{B^T B})$, and the optimization problem equivalent to the multi-output regression [3] with identity task covariance (herein $\mathrm{L}_1\mathrm{L}_{2,1}$G-solution). It is interesting to point out that, the regularization $\mathrm{Tr}(\mathbf{B^T B})$ is equivalent to imposing the matrix variate Gaussian priors on $(\mathbf{B^T B})^{1/2}$. From the definition of $\boldsymbol{\Omega}$, we have that $\boldsymbol{\Omega}_0^{-1} = \boldsymbol{\Sigma}_0$. This implies that $\boldsymbol{\Sigma}_0$ is PD as the covariance matrix from a multivariate Gaussian distribution. Therefore, a Cholesky factorization can be performed on $\boldsymbol{\Sigma}_0$ to obtain a lower triangular matrix $\mathbf{P}$ such that $\boldsymbol{\Sigma}_0 = \mathbf{P}\mathbf{P}^T$. By further defining $\mathbf{P} = \mathbf{U}_2 \boldsymbol{\Psi}_2 \mathbf{V}_2^T$ as the SVD of $\mathbf{P}$, we obtain

$$\boldsymbol{\Gamma}_1^T \boldsymbol{\Gamma}_1 (\mathbf{V}_1^T \mathbf{B} \mathbf{U_2}) + n\lambda_2 (\mathbf{V}_1^T \mathbf{B} \mathbf{U_2}) \boldsymbol{\Psi}_2 \boldsymbol{\Psi}_2^T = \mathbf{V}_1^T \mathbf{X}^T \mathbf{Y} \mathbf{U_2}. \tag{S6}$$

Using Eq (S6) and the change of variables

$$\begin{cases} \tilde{\mathbf{B}} & = \mathbf{V}_1^T \mathbf{B} \mathbf{U_2} \in \mathbb{R}^{n \times s} \\ \mathbf{S} & = \mathbf{V}_1^T \mathbf{X}^T \mathbf{Y} \mathbf{U_2} \in \mathbb{R}^{n \times s} \\ \boldsymbol{\Gamma}_1^T \boldsymbol{\Gamma}_1 & = \mathrm{diag}(\gamma_1, \gamma_2, \cdots, \gamma_n) \in \mathbb{R}^{n \times n} \\ \boldsymbol{\Psi}_2^T \boldsymbol{\Psi}_2 & = \mathrm{diag}(\psi_1, \psi_2, \cdots, \psi_s) \in \mathbb{R}^{s \times s}, \end{cases} \tag{S7}$$

one can see that for every row $1 \leq i \leq n$ and column $1 \leq j \leq s$, the entries of $\tilde{\mathbf{B}}$ can easily be computed as $\tilde{\mathbf{B}}_{i,j} = \dfrac{\mathbf{S}_{i,j}}{\gamma_i + n\lambda_2 \psi_j}$. From the change of variable $\tilde{\mathbf{B}} = \mathbf{V}_1^T \mathbf{B} \mathbf{U_2}$, we obtain the $\mathrm{L}_1\mathrm{L}_{2,1}$G-solution as $\mathbf{B} = \mathbf{V}_1 \tilde{\mathbf{B}} \mathbf{U}_2^T$ and refer to this as $\mathrm{L}_1\mathrm{L}_{2,1}$G-solution.

ii. When $\mathbf{C} = \mathbf{I}_s$ and $\boldsymbol{\Omega} = \mathbf{I}_s$, we get $\widehat{\mathbf{B}} = (\mathbf{X^T X} + n\lambda_2 \mathbf{I}_p)^{-1} \mathbf{X^T Y}$, the ridge estimate.

iii. When $\lambda_2 = 0$, we obtain $\widehat{\mathbf{B}} = (\mathbf{X^T X})^{-1} \mathbf{X^T Y}$, the ordinary least square estimate.

iv. When $\boldsymbol{\Omega} = \mathbf{I}_s$, we have the $\mathrm{L}_{2,1}$ feature selection estimate [4].

Method 3: Derivation of Ω as the solution to a special form of algebraic Riccati equation

Quadratic matrix equations of the form

$$\mathbf{AX^2} + \mathbf{BX} + \mathbf{C} = 0, \quad \mathbf{A}, \mathbf{B}, \mathbf{C} \in \mathbb{R}^{s\times s} \tag{S8}$$

can be solved explicitly by applying the usual formula for the roots of a scalar quadratic form if: (i) $\mathbf{A} = \mathbf{I}$, (ii) $\mathbf{B}$ commutes with $\mathbf{C}$, and (iii) $\mathbf{B^2} - 4\mathbf{C}$ has a square root [5, 6]. In what follows, we show that conditions (i), (ii) and (iii) are fulfilled for Eq (S9)

$$2\lambda_1\mathbf{\Omega^2} + \mathbf{P\Omega} - \mathbf{I}_s = 0, \tag{S9}$$

(***a***)- After rewriting Eq (S9) as $\mathbf{\Omega^2} + \frac{1}{2\lambda_1}\mathbf{P\Omega} - \frac{1}{2\lambda_1}\mathbf{I}_s = 0$, it becomes apparent that the following correspondence with respect to Eq (S8) can be established: $\mathbf{X} = \mathbf{\Omega}$, $\quad \mathbf{B} = \frac{1}{2\lambda_1}\mathbf{P}$, $\mathbf{C} = -\frac{1}{2\lambda_1}\mathbf{I}_s$ and $\mathbf{A} = \mathbf{I}_s$, which shows that (i) is satisfied.

(***b***)- Two matrices $\mathbf{M}$ and $\mathbf{N}$ with appropriate dimensions commute if and only if $\mathbf{MN} = \mathbf{NM}$. Hence, we need to show that $\left[\frac{1}{2\lambda_1}\mathbf{P}\right]\left[-\frac{1}{2\lambda_1}\mathbf{I}_s\right] = \left[-\frac{1}{2\lambda_1}\mathbf{I}_s\right]\left[\frac{1}{2\lambda_1}\mathbf{P}\right]$. The fact that one of the matrix involved in the product is the identity, and as such, commutes with all matrices of appropriate size, is sufficient to conclude that (ii) holds.

(***c***)- It is known that an $n \times n$ matrix $\mathbf{A}$ with n distinct non-zero eigenvalues has 2^n square roots. Such a matrix can be decomposed as $\mathbf{A} = \mathbf{VDV^{-1}}$, where $\mathbf{V}$ is a matrix with columns the eigenvectors of $\mathbf{A}$ and $\mathbf{D}$ a diagonal matrix with diagonal entries the eigenvalues of $\mathbf{A}$. Thus, $\sqrt{\mathbf{A}} = \mathbf{VD^{1/2}V^{-1}}$, with $\mathbf{D^{1/2}}$ any square root of $\mathbf{D}$. In addition, a non negative definite (i.e. either positive definite or semidefinite) matrix has exactly one non negative definite square root [7]. With this in mind, let

$$\mathbf{\Delta} = \mathbf{B}^2 - 4\mathbf{C} = \left[\frac{1}{2\lambda_1}\mathbf{P}\right]^2 + \left[\frac{2}{\lambda_1}\mathbf{I}_s\right]. \tag{S10}$$

(***c*₁**)- As the product of a matrix by its transpose, $\mathbf{P}$ is guaranteed to be symmetric positive semidefinite (PSD) (and positive definite if $\mathbf{Y} - \mathbf{XB_0}$ has linearly independent columns) and so must be $\mathbf{P}^2$.

(***c*₂**)- $\mathbf{\Delta}$ is PD as the sum of PSD and PD matrices.

($\boldsymbol{c_1}$) and ($\boldsymbol{c_2}$) imply that Eq (S10) has a unique positive definite square root, and hence (iii) holds.

Since conditions (i), (ii) and (iii) are satisfied, we conclude that the usual formula for the roots of scalar quadratic equations generalizes to the matrix counterpart in Eq (S8), and for our problem in Eq (S9) this leads to a unique solution defined by

$$\mathbf{\Omega}(\mathbf{B}_0) = \frac{1}{2\lambda_1}\left[(\mathbf{P}^2 + 8\lambda_1\mathbf{I}_s)^{\frac{1}{2}} - \mathbf{P}\right], \tag{S11}$$

where $\frac{1}{2\lambda_1}(\mathbf{P}^2 + 8\lambda_1\mathbf{I}_s)^{\frac{1}{2}}$ is the unique PD square root of $\mathbf{\Delta} = \mathbf{B}^2 - 4\mathbf{C}$.

References

1. Sylvester J. Sur la solution du cas le plus général des équations linéaires en quantités binaires, c'est-a-dire en quaternions ou en matrices du second ordre. CR Acad Sci Paris. 1884;99:117–118.

2. Tylavsky DJ, Sohie GRL. Generalization of the matrix inversion lemma. Proceedings of the IEEE. 1986;74(7):1050–1052. doi:https://doi.org/10.1109/PROC.1986.13587.

3. Cai H, Huang Z, Zhu X, Zhang Q, Li X. Multi-output regression with tag correlation analysis for effective image tagging. In: International Conference on Database Systems for Advanced Applications. Springer; 2014. p. 31–46.

4. Nie F, Huang H, Cai X, Ding CH. Efficient and robust feature selection via joint $l2$,1-norms minimization. In: Advances in neural information processing systems; 2010. p. 1813–1821.

5. Higham NJ. Computing real square roots of a real matrix. Linear Algebra and its applications. 1987;88:405–430. doi:https://doi.org/10.1016/0024-3795(87)90118-2.

6. Higham NJ, Kim HM. Numerical analysis of a quadratic matrix equation. IMA Journal of Numerical Analysis. 2000;20(4):499–519. doi:https://doi.org/10.1093/imanum/20.4.499.

7. Koeber M, Schäfer U. The unique square root of a positive semidefinite matrix. International Journal of Mathematical Education in Science and Technology. 2006;37(8):990–992. doi:https://doi.org/10.1080/00207390500285867.
