# Supplementary material for: Gene regulatory network inference using mixed-norms regularized multivariate model with covariance selection
Source: PLoS Comput Biol. 2023 Jul 31;19(7):e1010832. doi: 10.1371/journal.pcbi.1010832 (PMC10414675; doi:10.1371/journal.pcbi.1010832)
Supplement: Attachment — Submitted filename: Response to reviewers.docx [file pcbi.1010832.s005.docx]

**Response to editor and reviewers' comments to “Gene regulatory network inference using mixed-norms regularized multivariate model with covariance selection”**

All reviewers’ comments are replicated in blue font, and our point-by-point responses are provided in black font.

*Comments to the Authors:*

**Reviewer #1**:

*Gene regulatory networks are the underlying mechanism of controlling the expression of genes. Although lots of inference methods have been proposed to solve the problem, most of them ignore to analyze the characteristic of regulators and target genes. In this paper, the authors proposed two novel GRN reconstruction approaches to incorporate the L2,1-norm penalty with regularized multivariate regression and covariance selection. The experimental results on DREAM5 datasets show the good performance of the proposed method.*

**Response:** We thank the reviewer for this important point and for highlighting one of the contribution of our proposed approaches.

*However, there are some questions I concerned:*

1) *The application of L2,1-norm in reconstruction of gene regulatory networks is not a novel idea (similar idea in the following citation), so the authors need to compare them and clarity the main contribution. Gui S, et al. BMC bioinformatics, 2017, 18: 1-13.*

We agree with the reviewer that the L2,1-norm penalty has been previously used in different studies for GRN reconstruction. For instance, these include: (1) The PL21GPCA **[1]**, where the Lp-norm, the L2,1-norm and the Graph Laplacian PCA are combined to propose a dimensionality reduction method for gene network module discovery and robust tumour sample clustering. (2) The NMF-L2,1-norm **[2]** that uses nonnegative matrix factorization along with the L2,1-norm to select characteristic genes and the reference **[3]** suggested by the reviewer, to list a few. However, to the best of our knowledge, except the work in **[3]** these methods focus on feature selection rather than the inference of regulation between transcription factors (TF) and target genes (TG). Including these methods in our comparative analyses would have require additional assumptions (away from the original ideas of these studies) and extensive computation for proper model tuning.

Further, although the model by (Gui S, et al 2017) was in our primary list of models to include in the comparative analysis, we had to drop it because:_(1) It mainly focus on the inference of internal network structures using static or time-course data, (2) we wanted to use methods that have been previously applied on the similar data sets, and (3) we could not find enough information on how to choose their predefined upper bound regarding the number of incoming edges to a given gene for different data, thus leading to unfair comparison if these were included with random selection of values for this parameter.

2) *The authors should discuss the effects of time information on the construction of gene regulatory network, such as the time-delay effect on regulation.*

**Response:** We appreciate this important concern from the reviewer and acknowledge that time information and particularly the time-delay effect can be a very important aspect in the reconstruction of GRNs with data from time-course experiments. Even though challenging, the incorporation of time information can be particularly relevant when it comes to predict the system behaviour under different conditions, test hypotheses regarding the underlying regulatory mechanism and to shed more light onto our understanding of the dynamics of gene expression and cellular processes. In the manuscript, we applied the proposed approaches and the contenders on *E. coli* time-course data set without any usual time series modelling assumptions (e.g. stationarity, causality, homogeneity and time lag that can be understood as the maximum time delay). However, our models and some of the approaches in the comparative analysis could easily be tuned to fit the time series framework. While these assumptions are handy to simplify model building, the consideration of the specific biological system under investigation is equally important. We hope the reviewer will agree with us that, broadening the discussion around the concept of time-delay and its effects on GRN inference without making any use of models that directly account for it will not showcase the ability of those models to account for time-delay and will take the manuscript away from its original idea. Therefore, we maintain these points only in the response letter, and include a brief discussion on future research in the Conclusion.

3) *As single-cell transcriptome becomes popular in recent years, if the proposed method is also effective in scRNA-seq data?*

**Response:** We would like to thank the reviewer for bringing this important question to our attention. Our approach can be applied with any transcriptomics data, irrespective of whether or not they result from bulk or single cell profiling experiments. The reason is that the proposed approaches are built within a multivariate regression framework, so one only has to properly define the response and predictor variables that depends on the specific analysis of interest. For instance, our model could be used to infer the regulatory interactions between genes based on their expression levels in a population of cells (treated as samples). The response and predictor variables could then be the expression level of multiple target genes and potential regulatory genes respectively. An alternative would be to identify the key regulators of gene expression in specific cell types or cell subpopulations with response and the predictor variables as respectively the expression level of a group of specific genes in a cell type or subpopulation of interest and the expression levels of all genes in the scRNA-seq dataset. Finally, taking advantage of the pseudo-time ordering in scRNA-seq dataset and considering that the total number of time points is T, we can extend the work in **[4]** and hypothesize that the expression pattern of all genes at time point T is the total regulatory effect of the expression values of all genes at the previous (T-1) time points. However, a particular attention must be given on designing the ground truth (i.e. gold standard) for assessing the performance. Ongoing research use for instance, cell-type-specific ChIP-seq data, nonspecific ChIP-seq data and functional interaction network **[5]**. Because single-cell transcriptomic data have the potential to provide detailed information on the heterogeneity of gene expression within cells population, identify cell types, subpopulations, and regulatory networks at the single-cell level, we further investigated the identification of master regulators across different conditions, by employing the expression profiles of 10077 genes from ten independent GEO data sets with a total of 1118 non-small cell lung cancer (NSCLC) samples including both primary tumours (925 samples) and tumour-free control (193 samples) lung tissues **[6]**. This resulted in an additional section of results included in the updated version of the manuscript.

4) *The outputs of the proposed method are sparse networks, while the outputs of GENIE3 are the full networks, so the comparison of AUROC and AUPR are not so fair about the tail of ordered regulation relationships. The authors could use the early precision ratio instead.*

*Pratapa A, et al. Nature methods, 2020, 17(2): 147-154.*

**Response:** We acknowledge that using AUROC and AUPR to compare models with different level of sparsity may not be fair metrics. In addition to the early precision (EP) suggested by the reviewer, the comparative analysis has been extended to also include the normalized discounted cumulative gain (nDCG) **[7, 8]** that accounts for how early the true positive edges are found**.** The manuscript has been updated accordingly and we thank the reviewer for this important suggestion.

5) *Although the proposed method achieves the comparative performance than other non-linear ones (like GENIE3), the analysis of these two kinds of methods should be performed, such as the intersections of the results or advantages of these methods.*

**Response:** We thank the reviewer for bringing this key suggestion to our attention. We further recognise that performing this kind of analysis can provide valuable insights into the potential regulatory relationships between genes supported by both methods. By identifying common edges or interactions in the inferred GRNs, one can increase its confidence that these interactions are biologically meaningful and likely to represent true regulatory relationships. This can be particularly useful in situations where the individual GRN predictions generated by each method may have limited predictive power or suffer from a high degree of noise or false positives. In addition, this type of analysis can help in identifying which methods are most robust and reliable, as well as highlight areas of disagreement or uncertainty between different approaches. However, to draw meaningful biological conclusions, we think that such assessment should be performed in two steps and with several methods (*i.e.* 3 or more linear vs 3 or more non-linear). The first step and second steps will respectively study the within and between group intersection. Because of time limitation, to preserve the consistency of our manuscript, and to avoid drawing superficial conclusions, we opted not to add such analysis in the current manuscript and postpone it for a future study even though we understand the rationale behind the reviewer’s suggestion.

6) *Some recent citations are missing, such as :*

*[1] Moerman T, et al. Bioinformatics, 2019, 35(12): 2159-2161.*

*[2] Zheng R, et al. Bioinformatics, 2019, 35(11): 1893-1900.*

*[3] Shu H, et al. Nature Computational Science, 2021, 1(7): 491-501.*

**Response:** We agree that referencing pioneering efforts such as the ones suggested is crucial in providing the reader with a clear background regarding our current work and active research in the field. References have been appropriately provided in the updated manuscript and we thank the reviewer for this important suggestion.

**Reviewer #2**:

*The paper "Gene regulatory network inference using mixed-norms regularized multivariate model with covariance selection" by Mbebi and Nikoloski tries to quantify the power of joint methods (regularize multivariate regression and graphical models) in the classic task of reverse engineering gene regulatory networks (GRNs). In order to do so, the authors use data from DREAM5. Below, my points.*

- *An issue of the paper is that it uses data from two monocellular organisms, E.coli and S.cerevisiae. The task of GRN reconstruction has notoriously a step-up in obstacles when performed in higher eukaryotes. I suggest therefore that the authors apply their promising method in a higher eukaryote. The simplest task that comes to mind would be choosing a large dataset (e.g. a TCGA lung cancer dataset, which is publicly available and providing ~450 tumor samples plus 50 normal control samples). The optimal method for network reconstruction and master regulator definition (in the contrast tumor vs. normal) should be able to identify cancer genes (e.g. taken from the golden set at COSMIC Cancer Gene Census) with a higher precision (or accuracy) than simpler non-blended methods. Any other higher eukaryote dataset (e.g. a vertebrate, or even Drosophila) would be sufficient, the rationale behind this request being that E.coli and S.cerevisiae reconstruction is easier and has far less importance, due to the near-completion of regulatory networks in these species (e.g. in the RgulonDB project).*

**Response:** We thank the reviewer for this important point and for highlighting the use of joint methods that is in line with the usage we made of it in the manuscript. Further validation of master regulator identification across different tissues has been included in the updated manuscript. This has been achieved by analyzing a large expression profile data set comprising 10077 genes from 1118 non-small cell lung cancer (NSCLC) tissue samples of which 925 are affected by squamous cell carcinoma, adenocarcinoma and large cell carcinoma tumor, and 193 correspond to clinically healthy **[9]**. We wanted to evaluate the performance all contending methods in our comparative analyses, unfortunately, most of them are computationally intensive precluding comparative analyses even with the prolongation to the deadline for resubmission. However, for a fair comparison with recent promising method such PORTIA and it variant etePORTIA, we also considered the human lymphoblastoid cell lines (LCL) expression datasets **[10]**. The choice of this data set was motivated by an existing benchmarking with the contending approaches. We have updated the manuscript to account for these additional analyses and we hope the reviewer will understand our reasons for selecting these data sets**.**

- *The MR algorithms used by the authors use the common paradigm in GRN reconstruction literature, which briefly assumes that a TF-target edge is causally oriented from the TF to the target gene. It would be better if the authors clearly stated this causality assumption. Also, given the evident experience of the authors, they could discuss if their proposed inference approaches may benefit from causal algorithms to improve MR definition. This would improve the role of this paper in the development of a future generation of causal inference methods in Master Regulator Analysis.*

**Response:** We appreciate this concern from the reviewer which we have addressed in the original version of the manuscript but may not have been properly explained. Transcription factors (TFs) are key main regulators of gene transcription, and we implicitly assumed this to be understandable. However, to put things into more context and for a better flow, we have addressed this issue by clearly specifying in the updated manuscript that the mode of regulation is assumed to be oriented from transcription factor to target gene. Further, suggestion on how algorithms built for master regulator identification could integrate conditional independence as a pruning step has been included in the updated manuscript as well and we thank the reviewer for this suggestion.

- *The article is very technical and is hard to read for a biological audience. There is a general lack of clarity when presenting the performance of the proposed method vs. other methods, and an almost non-existent explanation of how the Master Regulator is evaluated. I would suggest presenting the results in a more widely intuitive way, for example clearly stating, in the main text tables, precision and accuracy of the proposed inference, based on different parameters of -for instance - the L2L2,1 norms models.*

**Response:** Additional explanations have been provided in the updated manuscript and we thank the reviewer for these constructive suggestions.

- *Connected to the previous point, the supplementary figures should definitely be moved to the main text (which currently has NO figure). This should be associated with a graphical improvement of the figures themselves. For example, Figure S1 should have higher resolution, thicker lines, and more clear color definition (for example with different combinations of colors and line types.*

**Response:** We appreciate this important concern from the reviewer and recognise that moving Figure S1 to the main text could improve the readability of our work. Further, we acknowledge that improving the quality of the figures will better highlight the differences or similarities in the comparative analysis. Where appropriate, figures have been restructured to showcase the differences wherever possible and the manuscript updated appropriately.

- *I am glad the authors provided their code on Github, however the code is currently irreproducible, as the authors didn't provide some of the raw data required to execute it. One example is in the file L1L21_Dream5_Scerevisia_example_run.R which at line 7 requires the non-existing file "net4_expression_data.tsv", and at line 10 "net4_transcription_factors.tsv". Until the code is made fully reproducible, including the generation of the figures in the text, I cannot critically evaluate its correctness.*

**Response:** We appreciate these concerns from the reviewer and agree that input data sets needed to reproduce example run are missing. The Github repository has been updated to include the required files. In addition, sample codes for figures generation have been provided as well.

**Reviewer #3**:

*The authors presented a new method for gene regulatory network (GRN) inference based on sparse linear regression and covariance matrix estimation. Four different models have been proposed, and benchmarked against state-of-art GRN inference techniques on the well-known DREAM5 data set. Because the gold standard networks from DREAM5 are mostly based on simulations, the authors also had to complement their work with real-world networks. In particular, they used time series transcriptomic data from a previous study on E. Coli, sampled across different tissues and experimental conditions. In terms of GRN reconstruction, the proposed method shows encouraging performance compared to already existing methods, especially given the simplicity and elegance of the model. Finally, they demonstrated how their approach can be used for identifying master regulators based on the sparsity patterns of the inferred weight matrices. The main contribution of this work mainly lies in the mathematical developments, which all prove to be correct. The manuscript is overall well-written and easy to read.*

*Major comments*

--------------

*Four inference approaches have been proposed, namely $L_1 L_{2, 1}$, $L_1 L_{2, 1}G$, $L_2 L_{2, 1}$ and $L_2 L_{2, 1}G$. Based on the text, it is pretty clear that $L_1 L_{2, 1}$ and $L_2 L_{2, 1}$ differ by the regularisation function used to penalise the precision matrix $\Omega$. However, I could not find what $G$ refers to, and what the 2 additional methods $L_1 L_{2, 1}G$ and $L_2 L_{2, 1}G$ consist in.*

**Response:** We thank the reviewer for summarising the main contributions of our work and for bringing to our attention this important point regarding the meaning of G in $L_1 L_{2, 1}G$ and $L_2 L_{2, 1}G$. To avoid having too much equations for the interested readers, we had to restructure the original manuscript whereby some methods were moved to the supplement including the part where the definition of G was mentioned. As shown in the supplementary material, the letter G is added on the main model because they are special cases corresponding to imposing a Gaussian prior on $(\bm{\mathrm{B^{T}B}})^{1/2}$. Thanks to the reviewer, we realised that we did not pay enough attention to preserve the manuscript’s flow in original submission. We have now double checked for any similar issues and addressed them where needed and updated the manuscript to be self-contained.

*Table 2 is suffering from severe shortcomings, as some of the methods present in table 1 are missing in table 2. The 4 proposed methods indeed seem to outperform Fused Lasso on the time-resolved transcriptomics data sets and Fused Lasso has already been applied on the same data in the past, but the publication dates back from 2016 and has never been compared to PORTIA, ENNET and PLSNET, among others. The authors should include all the contenders in table 2.*

**Response:** We have extended the table to including all methods in the comparative analysis in the updated manuscript and we thank the reviewer for this suggestion.

*While linear modelling of the relationship between transcription factors (TF) and target genes (TG) remains highly relevant (the use of non-linear models is not well motivated in the literature actually), the choice of using $L_{2, 1}$ regularisation still lacks motivation. The authors assumed that the number of TFs is considerably smaller than the number of TGs, and that each TF is likely to regulate many TGs. On synthetic data sets, this design choice might lead to poorer performance. For example, in DREAM3 and DREAM4 in silico networks, most genes regulate at least one TG each, and each regulates a few of them. Moreover, even in real-world settings, this can happen as a side-effect of gene pre-selection (e.g. based on a priori knowledge or quality-based filtering). Therefore, it is crucial that the authors clearly demonstrate the relevance of $L_{2, 1}$ regularisation, either by (1) further validating their models on additional data sets for which the gold standard networks do not have this idealised topology, or (2) illustrating through some basic graph-theoretical analysis or literature review that most gold standard networks actually reflect such row-wise sparsity patterns. Optionally, the authors could also comment on these limitations and provide recommendations on how to manually tune the hyper-parameter $\lambda_2$. Ideally, a script could be provided on the github repository for the automated tuning of $\lambda_2$ based on 10-fold cross-validation.*

**Response:** We appreciate these concerns from the reviewer which we have addressed in the original version of the manuscript but may not have been properly explained. For instance, it is clearly mentioned in the closing paragraph of the introduction that “the proposed models are combinations between regularized multivariate regression and graphical models in the large-*p*-small-*n* setting”. From the multivariate side, instead of regressing a single response variable on (*p>1)* predictors, we regress (*s>1)* responses on (*p>1)* predictors. For the type of analysis at hand, we deal with a special situation where we often have (with high probability) more responses than predictors, whereby all genes are considered as TGs until proven otherwise by experimental validation. In this scenario, the number of equations (response variables) exceeds the number of unknowns (predictor variables), and as a result, it is not possible to find a unique solution that perfectly satisfies all the equations simultaneously. However, by solving the proposed $L_2 L_{2, 1}$ optimization problem, we proved the existence and uniqueness of a positive definite solution via quadratic matrix equations. The idealised situation the reviewer is referring to (*e.g.* DREAM4 multifactorial networks) where the response and predictor are both square matrices, (*i.e.* balanced problem), does not fit with the second aspect of our proposed approaches (*i.e.* large-$p$-small-$n$ situation). Under this framework, methods such as entropy balancing, matrix balanced loss, multivariate ANOVA with Pillai's trace or even simple OLS could be more appropriate.

While acknowledging that these scenarios can indeed occur, we opted not to extend the discussion in that direction for the following reasons: (1) Assuming that the number of TFs is considerably smaller than the number of TGs, and that each TF is likely to regulate many TGs, is a supported **[11]** and commonly used assumption. (2) This will set an additional goal for the proposed manuscript, and (3) proper hyper parameter tuning needed to draw valid conclusions requires extensive computational time, which is prohibitive to report in the present study given the number of data sets we employed for the comparative analysis. Nevertheless, given the importance of the imbalance nature of GS often encountered in GRN inference and related to the other reviewer’s concern, we have included a sentence in the conclusion to indicate that future research is needed in designing efficient techniques for hyperparameters tuning. We have updated the manuscript accordingly and further motivation as to why the $L_{2, 1}$ was chosen are also provided and thank the reviewer for these constructive concerns.

*The authors reported that "all MR1 that the proposed L1L21 and L2L21 found conserved across all four conditions 231 have a maximum condition specificity index of .533". Is the inverse true as well? Are the genes with low specificity index listed among the genes selected by the models in all four conditions? Currently, table 3 does not give a good sense of the specificity and sensitivity of this approach. A more informative figure showing the distributions of all tau indexes would be valuable. Also, it is not clear from the text that there are only 3 MR1 TFs and 3 MR2 TFs (as suggested by the table). If more master regulators have been inferred by the proposed methods, I would recommend showing the whole list.*

**Response:** The identification of MR “conserved across conditions” is as follows: (1) a transcription factor is first identified as master regulator (type 1 or 2) for each individual condition, (2) we next select only those MR appearing in all four conditions, and (3) the tau index is used as a sanity check because if a MR is found conserved across conditions, then its expression level should be stable in all conditions. This means that a conserved MR might have a low index and not the other way around. We are aware that additional MR could be detected using different sparsity threshold and that the set of stably expressed genes from different approaches is not uniform. However, we fully understand the reviewer’s concern that is related to the way housekeeping genes are defined in the literature. To this end, we further tested our methodology on NSCLC data sets to identify MR conserved across tumour and healthy tissues. A similar conclusion was reached, whereby some master transcription factor genes had a low specificity index despite not identified to be conserved across tissues. We updated the manuscript to include a discussion as to why this could happen and we would like to thank the reviewer for this important question.

*Also, the presence of 0 tau indexes for genes alaS and flhC is surprising. In the original paper where the tau index presented in equation 17 was introduced, the expression values $x_i$ are supposed to be normalised by the maximum. This should be mentionned in the text for clarity purposes, because it is unclear whether the maximum value has been computed across genes or samples. Assuming that the authors normalised across genes, then based on equation 17 this would mean that alaS and flhC both had the highest expression value, and these values were the same in all experimental settings. This is extremely unlikely, so I assume the authors normalised across samples. However, this still means that these 2 genes had constant expression, which is unlikely as well. Could the authors comment on that? Overall, I think table 3 and the corresponding section should be revised in order to clarify all these points.*

**Response:**  After carefully reviewing the source code in the R package “tispec” we previously used, we realized that the specificity implemented in the package was different than the reference in our manuscript. We have updated the manuscript to account for the proper formula in the original article where the tau index presented in Eq.(17) was introduced. Further, the gene expression data have been normalized by the maximum expression in all four considered conditions. We have restructured the table and updated the manuscript accordingly and we would like to thank the reviewer for bringing this important remark to our attention.

*While the manuscript is backed by an open-source implementation of each of the 4 models on the github repository, I would recommend adding standalone scripts to produce the results shown in tables 1 and 2. In particular, I did not find the code necessary to run their models on the time-resolved E. Coli data sets for GRN reconstruction and estimation of the tau indexes.*

**Response:** The Github repository has been updated to account for this suggestion and we thank the reviewer.

*Given the high level of technical details present in the paper, by contrast the solution presented in equation 9 is lacking context and developments. It is the only equation that cannot be deduced just by reading the text and without relying on external resources. I would recommend adding more developments to it in suppl. mat.*

**Response:** This has been dealt with in the updated manuscript, and we thank the reviewer for this important comment.

***Minor comments***

--------------

*Type-2 master regulators have been defined based on the sparsity of the weight matrix and the "non-zero TF-TG entries in the sparse precision matrix". Since the precision matrix is of shape TG x TG, the authors are implicitely assuming that the set of TGs necessarily contains the set of TFs. This could be made more explicit in the text.*

**Response:** This has been addressed in the updated manuscript and we thank the reviewer for this suggestion.

*The Sylvester equation (eq. 8) is not homogeneous, due to the independent term $X^T Y$ being different from 0. Same remark for suppl. eq. S1.*

**Response:** Indeed, the equation is inhomogeneous. Appropriate changes have been made in the updated version and we thank the reviewer for spotting this.

*There are minor tense inconsistencies, like "We use the estimated sparse regression coefficient" or "we use three benchmark data sets from the DREAM5", when most of the manuscript is written in the past tense.*

**Response:** We appreciate this valuable feedback from the reviewer. Consistency has been checked and the manuscript updated accordingly.

*"numner of TGs" -> "number of TGs"*

**Response:** This has been dealt with in the updated manuscript, and we thank the reviewer for this important comment.

*Conclusion*

*----------*

*Based on the simplicity, elegance, but also the novelty of the proposed approaches and the good performance shown on the DREAM5 benchmark, I would recommend making major revisions to the current manuscript. All the contenders listed in table 1 should be present in table 2 to allow a fair comparison. Table 3 and section about master regulator identification should be deeply revised to make it clear that the agreement between inferred master regulators and tau indexes is not due to random chance. Finally, the authors should investigate the relevance of their modelling choices (e.g. $L_{2, 1}$ regularisation) in other settings than the ones tested so far (e.g. DREAM5), and discuss how the topology of gold standard GRNs can affect the inference accuracy.*

________________________________

**Reference**

1) Kong, Xiang-Zhen, Yu Song, Jin-Xing Liu, Chun-Hou Zheng, Sha-Sha Yuan, Juan Wang, and Ling-Yun Dai. "Joint Lp-Norm and L2, 1-Norm Constrained Graph Laplacian PCA for Robust Tumor Sample Clustering and Gene Network Module Discovery." *Frontiers in Genetics* 12 (2021): 621317.
2) Wang, Dong, Jin-Xing Liu, Ying-Lian Gao, Jiguo Yu, Chun-Hou Zheng, and Yong Xu. "An NMF-L2, 1-norm constraint method for characteristic gene selection." *PloS one* 11, no. 7 (2016): e0158494.
3) Gui, Shupeng, Andrew P. Rice, Rui Chen, Liang Wu, Ji Liu, and Hongyu Miao. "A scalable algorithm for structure identification of complex gene regulatory network from temporal expression data." *BMC bioinformatics* 18 (2017): 1-13.
4) Gan, Yanglan, Xin Hu, Guobing Zou, Cairong Yan, and Guangwei Xu. "Inferring gene regulatory networks from single-cell transcriptomic data using bidirectional rnn." *Frontiers in Oncology* 12 (2022).
5) Guan, Jinting, Yang Wang, Yongjie Wang, Yan Zhuang, and Guoli Ji. "SRGS: sparse partial least squares-based recursive gene selection for gene regulatory network inference." *BMC genomics* 23, no. 1 (2022): 1-11.
6) Lim, Su Bin, Swee Jin Tan, Wan-Teck Lim, and Chwee Teck Lim. "A merged lung cancer transcriptome dataset for clinical predictive modeling." *Scientific data* 5, no. 1 (2018): 1-8.
7) Järvelin, Kalervo, and Jaana Kekäläinen. "Cumulated gain-based evaluation of IR techniques." *ACM Transactions on Information Systems (TOIS)* 20, no. 4 (2002): 422-446.
8) Walker, Angelica M., Ashley Cliff, Jonathon Romero, Manesh B. Shah, Piet Jones, Joao Gabriel Felipe Machado Gazolla, Daniel A. Jacobson, and David Kainer. "Evaluating the performance of random forest and iterative random forest based methods when applied to gene expression data." *Computational and Structural Biotechnology Journal* 20 (2022): 3372-3386.
9) Lim, Su Bin, Swee Jin Tan, Wan-Teck Lim, and Chwee Teck Lim. "A merged lung cancer transcriptome dataset for clinical predictive modeling." *Scientific data* 5, no. 1 (2018): 1-8.
10) Niu, Nifang, Yuxin Qin, Brooke L. Fridley, Junmei Hou, Krishna R. Kalari, Minjia Zhu, Tse-Yu Wu, Gregory D. Jenkins, Anthony Batzler, and Liewei Wang. "Radiation pharmacogenomics: a genome-wide association approach to identify radiation response biomarkers using human lymphoblastoid cell lines." *Genome research* 20, no. 11 (2010): 1482-1492.
11) Erwin, Douglas H., and Eric H. Davidson. "The evolution of hierarchical gene regulatory networks." *Nature Reviews Genetics* 10, no. 2 (2009): 141-148.
